# AC010973.2 promotes cell proliferation and is one of six stemness-related genes that predict overall survival of renal clear cell carcinoma

**DOI:** 10.1038/s41598-022-07070-1

**Published:** 2022-03-11

**Authors:** Yingqing Liu, Jiawei Wang, Lin Li, Haibo Qin, Yuang Wei, Xu Zhang, Xiaohan Ren, Wei Ding, Xudong Shen, Guangyao Li, Zhongwen Lu, Dong Zhang, Chao Qin, Lingsong Tao, Xinglin Chen

**Affiliations:** 1 Department of Urology, The Second People’s Hospital of Wuhu, Wuhu, 241000 Anhui Province China; 2 Department of Urology, The First Affiliated Hospital of Nanjing Medical University, Nanjing, 210009 China

**Keywords:** Biotechnology, Cancer, Computational biology and bioinformatics, Genetics, Molecular biology, Nephrology

## Abstract

Extensive research indicates that tumor stemness promotes tumor progression. Nonetheless, the underlying roles of stemness-related genes in renal clear cell carcinoma (ccRCC) are unclear. Data used in bioinformatics analysis were downloaded from The Cancer Genome Atlas (TCGA) database. Moreover, the R software, SPSS, and GraphPad Prism 8 were used for mapping and statistical analysis. First, the stemness index of each patient was quantified using a machine learning algorithm. Subsequently, the differentially expressed genes between high and low stemness index were identified as stemness-related genes. Based on these genes, a stable and effective prognostic model was identified to predict the overall survival of patients using a random forest algorithm (Training cohort; 1-year AUC: 0.67; 3-year AUC: 0.79; 5-year AUC: 0.73; Validation cohort; 1-year AUC: 0.66; 3-year AUC: 0.71; 5-year AUC: 0.7). The model genes comprised *AC010973.2*, *RNU6-125P*, *AP001209.2*, *Z98885.1*, *KDM5C-IT1,* and *AL021368.3*. Due to its highest importance evaluated by randomforst analysis, the *AC010973.2* gene was selected for further research. In vitro experiments demonstrated that *AC010973.2* is highly expressed in ccRCC tissue and cell lines. Meanwhile, its knockdown could significantly inhibit the proliferation of ccRCC cells based on colony formation and CCK8 assays. In summary, our findings reveal that the stemness-related gene *AC01097.3* is closely associated with the survival of patients. Besides, it remarkably promotes cell proliferation in ccRCC, hence a novel potential therapeutic target.

## Introduction

Renal cell carcinoma (RCC) is the most prevalent type of kidney cancer. It has > 10 histological and molecular subtypes; out of these, clear cell renal cell carcinoma (ccRCC) is the common form, approximately accounting for 75–80% and an incidence of > 5%. Moreover, it has frequent metastasis and poor prognosis^1^. The remaining subtypes, i.e., papillary RCC, chromophobe RCC, and unclassified RCC comprise non-clear cell RCC (nccRCC). Notably, nearly 92% of ccRCC carries the inactivation or mutation of the von Hippel-Lindau (VHL) gene located on human chromosome 3p, which causes highly vascularized and lipogenic characteristics of tumor tissues^2^. Early diagnosed renal cancers effectively benefit from partial nephrectomy (PN). Stereotactic ablative radiotherapy has been emerging as a treatment option for medically inoperable patients with localized cT1a and cT1b tumors. However, collectively, the prognosis of metastatic ccRCC is still unsatisfactory for its low benefit deriving from chemotherapy or radiotherapy; also, few patients show enough response to immunotherapy^3^.

Cancer stem cell (CSC) is a type of cell with continuously self-renewing capacity and generates differentiated cells in malignant tumors. In the process of neoplasia, specific single cells in tissues hierarchically grow through various genetic and epigenetic modifications, generate the evolution of pre-malignant subpopulations, and display malignant features as cancer cells^4,5^. CSCs are essential for aberrant proliferation, survival, invasion, and metastasis of tumors, that is why they have also been indicated as ‘tumor-initiating cells’ following the result of Working Conference on CSCs^6^. In ccRCC, accumulating evidence suggests a correlation between tumor progression, stemness-related pathways, and the pivotal role of CSCs in initiating the derivation of malignant subpopulations^7^. Previous studies indicate that a few biomarkers and chemokines are relevant to tumorigenesis and prognosis of renal cancer. Elsewhere, Yu et al. found that patients with higher *NANOG* and *OCT4* expression in tumor tissues had a lower survival rate^8^. Furthermore, Arezoo and colleagues found that *OCT4* and *NANOG* co-expression in renal cancer is significantly associated with RCC subtypes and predicted poor prognosis of ccRCC patients; also, they evaluated *CXCR4* as a novel renal CSC marker in renal cancer through immunohistochemistry (IHC)^9,10^. Using a similar technique, Kyungeun et al. confirmed that *CD133* is a favorable prognostic CSC biomarker for ccRCC^11^. Nevertheless, in addition to histological methods identifying potential biomarkers, integrative strategies have been hardly used to explore the mechanisms by which CSCs link to ccRCC.

Herein, we performed comprehensive bioinformatic analysis and molecular research to evaluate the bio function and prognostic value of stemness-associated genes in ccRCC. Using multiple databases and ccRCC cell lines, our findings ascertained the prognostic and oncogenic role of *AC010973.2* in ccRCC.

## Methods

### Public data acquisition and tumor stemness calculation

Expression profile and clinical information of ccRCC patients were downloaded from the TCGA database (TCGA-KIRC), including 72 normal and 539 tumor tissues. Data were preprocessed through the following steps: data merging and normalization, probe annotation, missing value completion. In detail, the raw data downloaded from TCGA database were annotated according to the genome reference file “Homo_sapiens.GRCh38.104.gtf”. Next, the curated expression profile was complemented with missing values and log2 transformed to eliminate the effect of extreme values. Also, the “Homo_sapiens.GRCh38.104.gtf” file was used to distinguish coding proteins and non-coding lncRNAs. The one-class logistic regression machine learning algorithm (OCLR) was used to obtain gene expression-based stemness index (mRNAsi) and epigenetic regulation based-stemness index (EREG-mRNAsi)^12^. The data used for OCLR was obtained from previous study and has been uploaded in the figshare (https://figshare.com/articles/dataset/Tumor_stemness_OLCR/16866574). Based on the calculated stemness value, all patients were divided into high and low stemness groups. The limma package was used to identify differentially expressed genes (DEGs) between high and low stemness groups with the threshold of |logFC > 1| and p-value < 0.05. These DEGs were defined as stemness-related genes.

### Gene enrichment analysis and protein interaction

Gene ontology (GO) analysis was performed using a clusterprofiler package to explore the underlying biological role of genes, comprising three terms, i.e., biological process (BP), cellular component (CC), and molecular function (MF). Gene set enrichment analysis was conducted to compare the difference in biological pathways between the two groups. The reference gene set was “Hallmark.7.4.symbol.gmt”. In detail, the number of permutations was set as 1000; the metric for ranking genes was set as “Signal2Noise”; the normalization mode was set as “meandiv”; the randomization mode was set as “no_balance”. Protein interaction of identified genes was performed in the STRING online database and visualized using the Cytoscape v3.7.2 software. Cytohubba plug-in was used to calculate the importance of all nodes and identify the key nodes.

### Construction of prognostic prediction model based on stemness-related genes

All the patients were randomly divided into training and validation groups. Univariate cox regression analysis was performed to identify prognosis-related genes. Then, dimensionality reduction was computed using a random forest algorithm with ntree = 1000. Each gene was allocated a relative importance value. Based on the top 6 important genes, multivariate cox regression analysis was conducted to construct a prognostic prediction model. All patients included in the prognosis model were assigned a risk score value. Then, the Receiver operating characteristic (ROC) curve was used to assess the stability of the prognosis model. The area under the roc curve (AUC) value > 0.7 was considered to have good predictive capabilities. The Kaplan–Meier survival curves were used to compare the prognostic difference between the two groups.

### Nomogram, decision curve analysis (DCA), and calibration curves

A nomogram was established after integrating the clinical features and the prognostic model using the nomogramEx package in the R software. DCA curve was plotted using the rmda package to evaluate the performance of the nomogram. The calibration curve was used to display the difference between actual and predicted survival.

### Real-time (RT) qPCR of tissue and cells

The 13 paired ccRCC and normal tissues were obtained from the first affiliated hospital of Nanjing Medical University. All patients signed informed consent. In total, four renal carcinoma cell lines (786-O, *ACHN*, *Caki-1*, *Caki-2*) and one normal kidney epithelial cells (HK-2) were purchased from the Cell Bank of the Chinese Academy of Sciences (Shanghai, China). Total RNA was extracted from tissue and cells using an RNA extraction kit (DP419, TIANGEN, Beijing, China) following the protocol. Total RNA was reversely transcribed to cDNA using a reverse transcription kit (TaqMan). Then, RT-qPCR was performed using SYBR Green methods (Applied Biosystems, Foster City, CA, USA). The primers used were included. AC010973.2, forward: 5′-TTCCGTTACAGAGCAAAACCT-3′; AC010973.2, reverse: 5′-ACCTCAGGGAACCTTGGATG-3′; GAPDH, forward: 5′-AC CACAGTCCATGCCATCAC-3′; and GAPDH, reverse: 5′-TCCACCACCCTG TTGCTGTA-3′.

### Western blotting

Western blot was performed as previously described^13^. Cells were seeded into a 10 cm dishes and grown to ∼90% density. Then, cells were added with RIPA lysis buffer (Beyotime) and lysed on ice for 2 h. Cell lysates were stored overnight at − 80 °C and cleared by centrifugation (20,000×*g*, 20 min, 4 °C). Protein lysates were boiled in loading buffer for 5 min. The proteins were visualized by ECL solution and captured by Quantity One V4.31. The primary antibody of cleaved caspase-3, Bcl-2, Bax, and GAPDH were purchased from Sigma.

### RNA interference

RNA interference was performed as previously described using small interfering RNA (siRNA)^13^. Lipofectamine 3000 (Invitrogen) was used to help transfecting siRNA-AC010973.2 into target cells. qPCR was used to evaluate the efficiency of siRNA interference. The targeting sequence used for siRNA against AC010973.2 was: siRNA1, 5′-GCTGTCTACAGAATAAACT-3′; siRNA2, 5′-CAGCCCTTATCGTCCATGA-3′; siRNA3, 5′-AGGCAGACGGGAGGGCTTA-3′.

### Colony formation assay

Colony formation assay was conducted as previously described^13^. The AC010973.2-knockdown cells were seeded into six-well plates with 500 cells per well. The cells were cultured for 7–14 days. Lastly, they were fixed with 4% formaldehyde for 15 min and then stained with crystal violet. The number of colonies was counted to evaluate the clonogenic capability of cancer cells.

### CCK8 assay

CCK8 assay was performed using Cell Couting Kit-8 (CCK8, Dojindo, Shanghai, China). Briefly, the AC010973.2-knockdown cells were seeded into 96-well plates with 2000 cells per well. Then, 10 μL CCK8 was added into the wells in a light-avoidance environment. Thereafter, they were maintained in an incubator at 37 °C and 5% CO2 for 1.5 h. Finally, the absorbance at 450 nm was detected to determine the proliferative ability of cancer cells.

### Statistical analysis

All analyses were conducted using the R software v4.0.0, SPSS v13.0, and GraphPad Prism 8. p-value was two-sided and < 0.05 was considered statistically significant. Student T-test was used to compare the difference between the two groups.

### Ethical approval

The studies involving human participants were reviewed and approved by the Ethics Committee of Jiangsu Province Hospital (approval number 2019-SR-125). The patients/participants provided their written informed consent to participate in this study. Only the data was collected from the database about the patients and all authors have declared that they agree to publish. all methods were performed in accordance with relevant guidelines and regulations.

### Informed consent

Informed consent was obtained from all individual participants included in the study.

## Results

### Identification of differentially expressed tumor stemness-related genes

**Figure 1:**
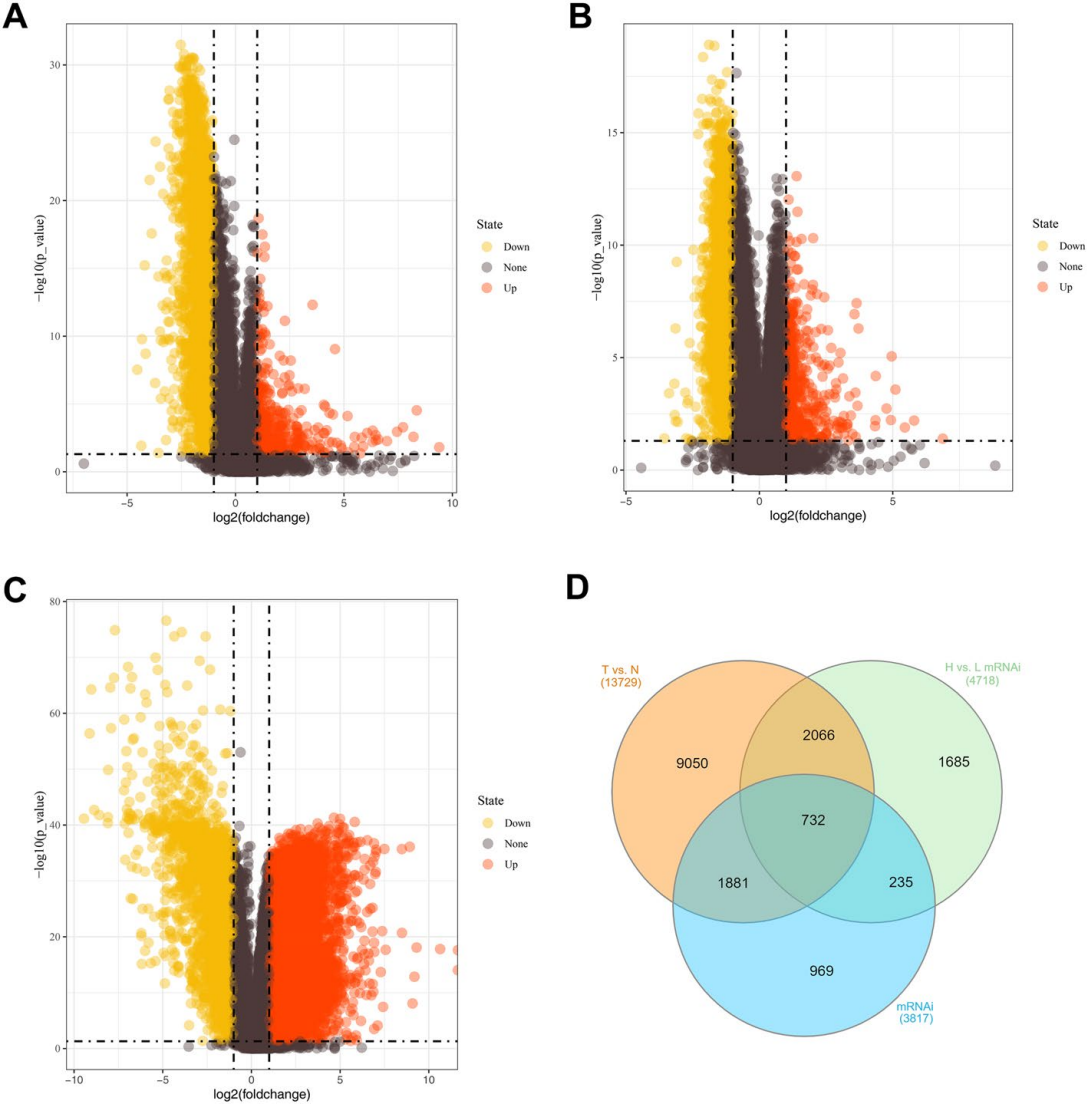
Identification of stemness indices-related DEGs. Notes: Volcano plots for DEGs in high-mRNAsi and low-mRNAsi ccRCC samples from (**A**), high-EREG-mRNAsi and low-EREG-mRNAsi ccRCC samples from (**B**), and tumor and normal tissues (**C**). (**D**) Venn diagram for DEGs from 3 different data sets. DEGs: differentially expressed genes; ccRCC: clear cell renal cell carcinoma.

Using the cutoff of |logFC|> 1 and the p-value < 0.05, 13,729, 4,718, and 3,817 differentially expressed genes (DEGs) were obtained via differential expression analysis on high and low mRNAsi groups (Fig. 1A), high and low EREG-mRNAsi groups (Fig. 1B), tumor and normal groups (Fig. 1C), respectively. After intersecting DEGs from three ccRCC-related datasets, 732 tumor stemness-associated DEGs in ccRCC were eventually identified (Fig. 1D).

### Function and interaction analysis of tumor stemness-related DEGs

**Figure 2:**
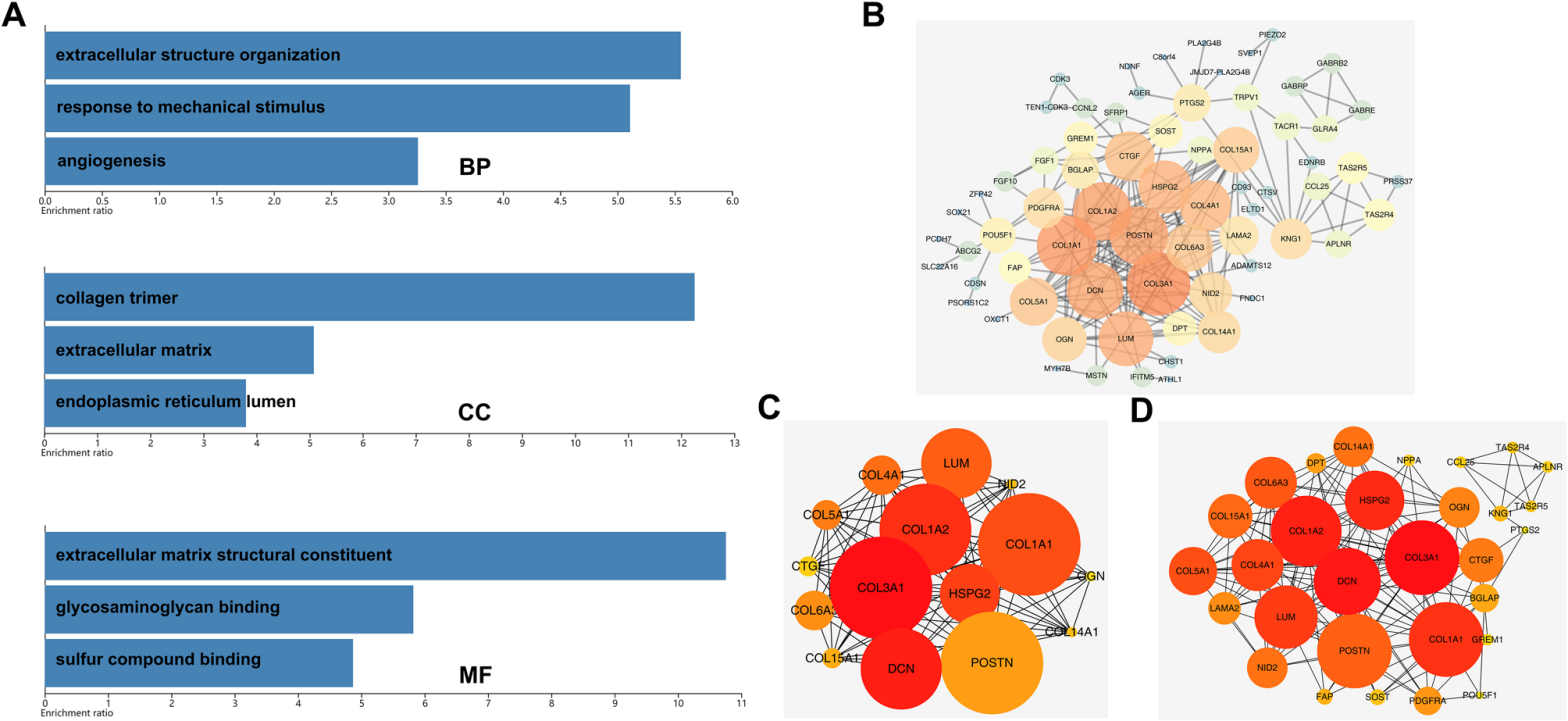
Function analysis on DEGs. Notes: (**A**) GO enrichment analysis of 732 DEGs, BP: biological process; CC: cell component; MF: molecular function. (**B**) PPI network of 732 DEGs after removing nodes without edges. (**C**) Top 30 nodes of PPI network. (**D**) Top 15 nodes of PPI network. GO: gene ontology; PPI: protein–protein interaction.

Gene ontology (GO) enrichment analysis and protein–protein interaction (PPI) analysis were conducted to explore the potential biofunction of 732 stemness-related DEGs in ccRCC and unravel the intrinsic roles of these genes. GO annotation analysis revealed that DEGs were tightly associated with the construction of extracellular matrix (ECM) structure in respect of cellular component, molecular function, and biological process (Fig. 2A). By calculating the score of nodes, the most significant modules with 15 nodes (Fig. 2C) and 30 nodes (Fig. 2D) from the PPI network (Fig. 2B) showed potential hub genes and interactions between them.

### Construction, evaluation, and validation of the stemness indices-based 6-gene risk signature

**Figure 3:**
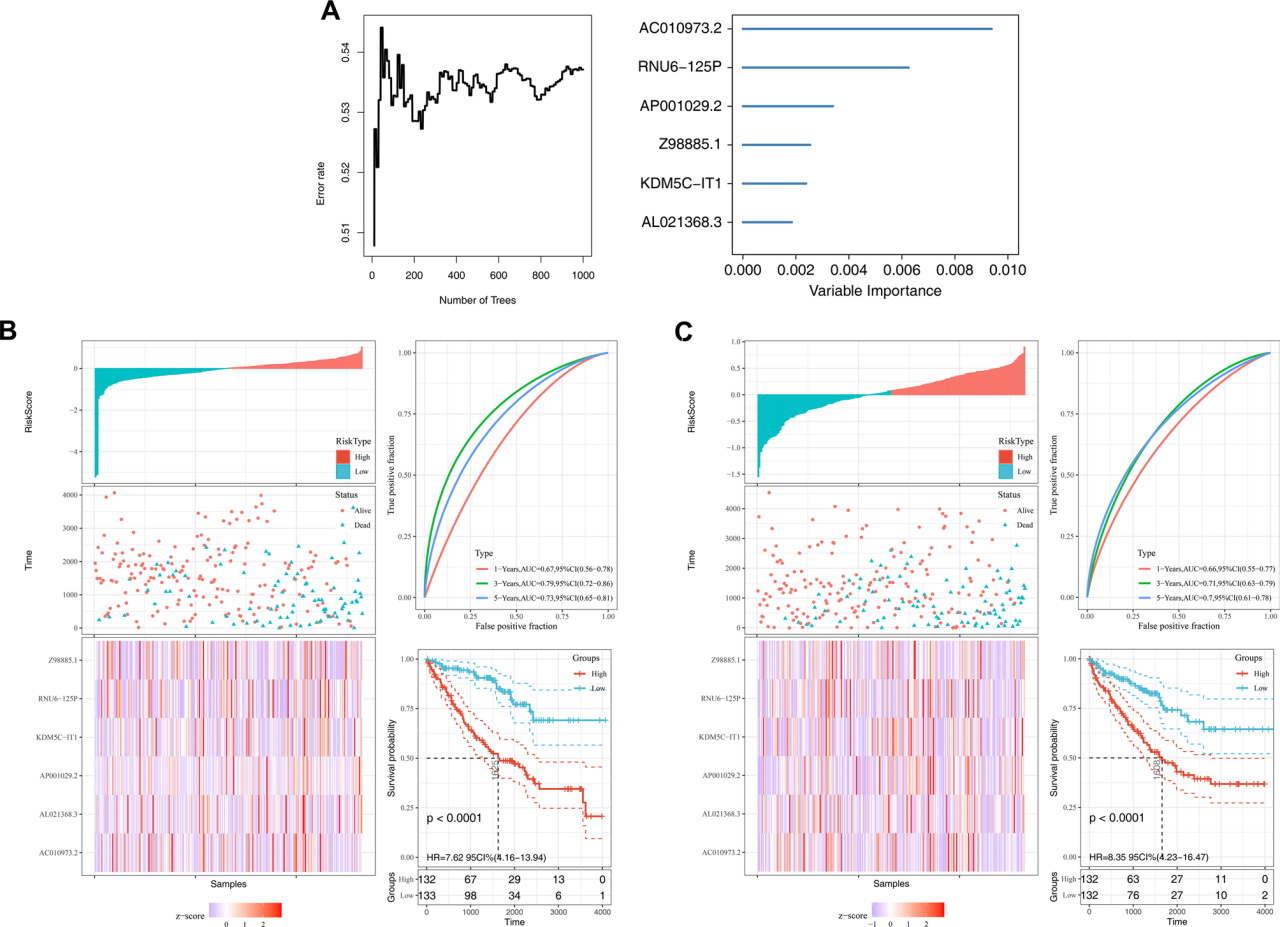
Establishment and assessment of the risk signature for ccRCC. Notes: (**A)** Calculation of relative importance of each DEG in the prognosis of ccRCC patients using Random Forest algorithm. In (**B**) Training cohort and (**C**) Validation cohort, diagrams are risk score map (left top), survival status map (left medium), gene expression heat map (left bottom), time‐dependent ROC curves (right top) and Kaplan–Meier survival curves (right bottom). ROC: receiver operating characteristics.

A total of 529 ccRCC patients with complete follow-up information in the TCGA-KIRC database were randomly divided into training and validation cohorts at a ratio of 1:1 to establish a prognostic prediction model for ccRCC patients based on the stemness indices (mRNAsi and EREG-mRNAsi). First, univariate cox analysis was performed to identify prognosis-related genes with a p-value threshold < 0.05. Then, Random Forest (RF) algorithm was applied to minimize the dimension and calculate the relative importance score of these prognosis-related genes. Consequently, each gene was given a score and the top six genes with the highest scores were associated with the OS of patients (Fig. 3A). Then, they were used to establish a risk signature for the prognosis of ccRCC patients after multivariate cox analysis. In the 6-gene risk signature, the “∑coef ∗ Exp(genes)” formula used to calculate the risk score for each patient was: Risk Score = 0.133 * AC010973.2 expression + 0.182 * AP001029.2 expression + (−0.271) * RNU6-125P expression + (−0.025) * Z98885.1 + (−0.114) * KDM5C-IT1 expression + (−0.175) * AL021368.3. Based on the median risk score in the signature, patients from training (Fig. 3B) and validation (Fig. 3C) cohorts were equally divided into low‐risk and high‐risk groups. Diagrams of risk score (left top), survival status (left medium), the heat map of gene expression (left bottom), time‐dependent ROC curves (right-top), and Kaplan–Meier survival curves (right bottom) are shown in the figures. Time-dependent ROC curves showed AUC of the prognostic 6-gene signature of 0.67 at 1 year; 0.79 at 3 years and 0.73 at 5 years in the training cohort (Fig. 3B), and a similar outcome of 0.66 at 1 year, 0.71 at 3 years, and 0.7 at 5 years in the validation cohort (Fig. 3C). Survival analysis revealed a huge advantage OS of the patient in the low-risk group compared to the high-risk group both in training (p < 0.0001, Fig. 3B) and validation groups (p < 0.0001, Fig. 3C).

### Clinical parameters-based model and comparison between two prognosis prediction models

**Figure 4:**
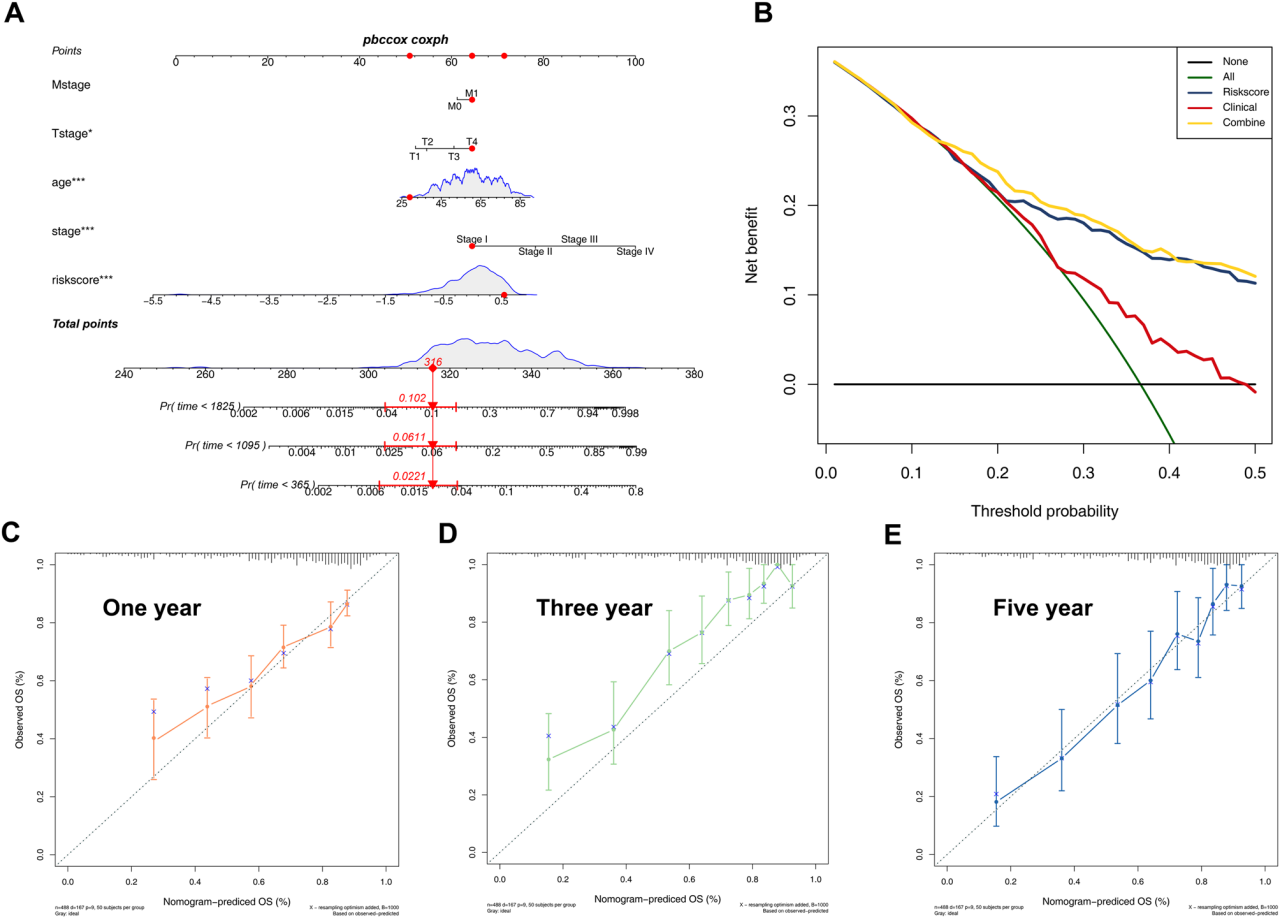
Decision Curves Analysis (DCA) of clinical model and risk signature. Notes: (**A**) Nomogram of clinical parameters-based prediction model for prognosis of patients. (**B**) Decision curves for different predicting models. Horizontal line: assume all patients as low risk (Pi < Pr); Green curve: assume all patients as high risk (Pi > Pr); Red curve: clinical prediction model; blue curve: gene signature; Yellow curve: combination use of two models. Calibration curves for nomogram in predicting survival probability at 1 year, 3 years and 5 years. The calibration measurement was conducted through bootstrapping 1000 resamples in TCGA-KIRC database (**C-E**).

By assigning the OS as the outcome, a nomogram was constructed based on the clinical features and risk score of ccRCC patients (Fig. 4A). As shown in Fig. 4A, age (p < 0.001), stage (p < 0.001), T stage (p < 0.05), and risk score (p < 0.001) revealed a significant relationship with OS. To compare the capacity of the clinical prediction model and 6-gene signature in prognostic prediction for ccRCC patients, decision curves analysis (DCA) was conducted by labeling model of risk signature as ‘Riskscore’, clinical prediction model as ‘Clinical’ and combination of two models as ‘Combined’ (Fig. 4B). A noticeable distinction of Net benefit (NB) between ‘Riskscore’ and ‘Clinical’ curves were noted when threshold probability (Pt) ranged from 0.2 to 0.5, suggesting the superiority of gene signature over the clinical model bringing medical benefits for the whole cohort under prediction. By comparing the blue and yellow curves, we confirmed the predictive capacity of the combined model slightly preceded the gene signature alone. Assessment of the model for efficacy in predicting 1-year (Fig. 4C), 3-year (Fig. 4D), and 5-year (Fig. 4E) survival probability was performed by measuring calibration through bootstrapping 1000 resamples (Gray = ideal).

### Clinical relevance and potential bio-functional pathways of hub gene *AC010973.2*

**Figure 5:**
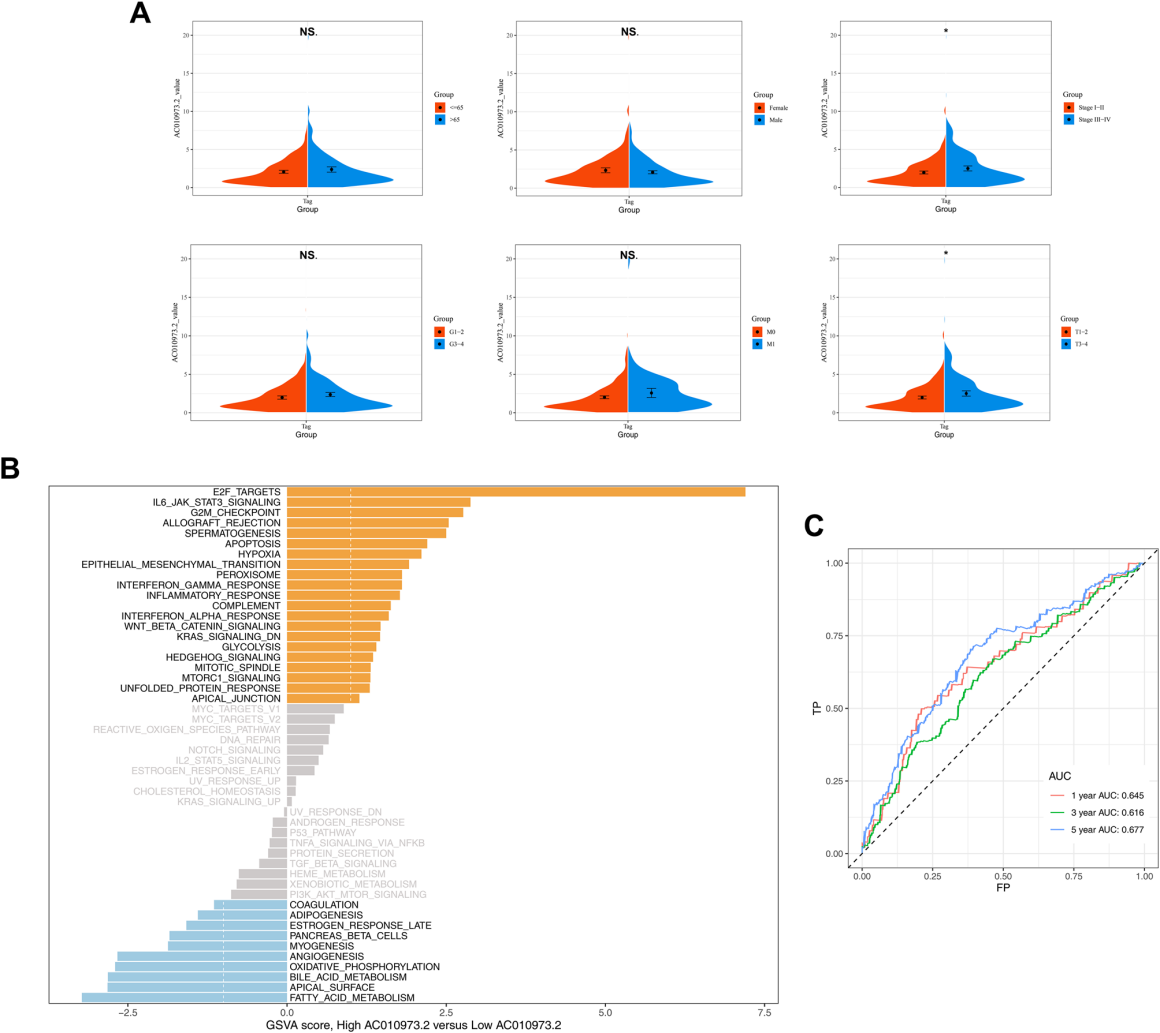
Clinical correlation analysis and GSVA of AC010973.2. Notes: (**A**) Violin plots for distribution of AC010973.2 value in different groups of diverse clinicopathological features in XXX patients from KIRC database. (**B**) Diagram of GSVA on AC010973.2. The collection used was H-hallmark gene sets. Y-axis represented diverse hallmark gene sets. X-axis represented gene sets expression level in high (yellow bars) and low (blue bars) AC010973.2 groups. (**C**) ROC curves for AC010973.2 alone to predict 1-year, 3-year and 5-year OS of ccRCC patients. AUC for them were exhibited in the plot.

*AC010973.2* gene was selected for further research because of its significance in the risk signature (Fig. 3A). Clinical correlation analysis demonstrated no meaningful relationships between *AC010973.2* expression and age, gender, pathological grade, and M stage. Nevertheless, the *AC010973.2* value was significantly upregulated in individual stage III-IV compared to stage I-II (p < 0.05), and in T stage III-IV compared to T stage I-II (p < 0.05) (Fig. 5A). The results revealed that *AC010973.2* potentially participates in the proliferation and growth of the tumor as a pro-cancer gene.

To explore the associated pathways involving *AC010973.2*, Gene Set Variation Analysis (GSVA) analysis was used to detect signature differentiation between high and low expression groups. Several representative cancer pathways were tightly related to *AC010973.2*, including E2F targets, IL6-JAK-STAT3 signaling, apoptosis signaling, hypoxia signaling, and fatty acid metabolism (Fig. 5B). Further, *AC010973.2* was applied as an independent predictor for the OS of patients in ccRCC; the ROC curves showed the AUC of 0.645, 0.616, and 0.677 for survival probability at one year, three years, and five years, respectively (Fig. 5C).

### Upregulation of *AC010973.2* expression in ccRCC tissues and cell lines

**Figure 6:**
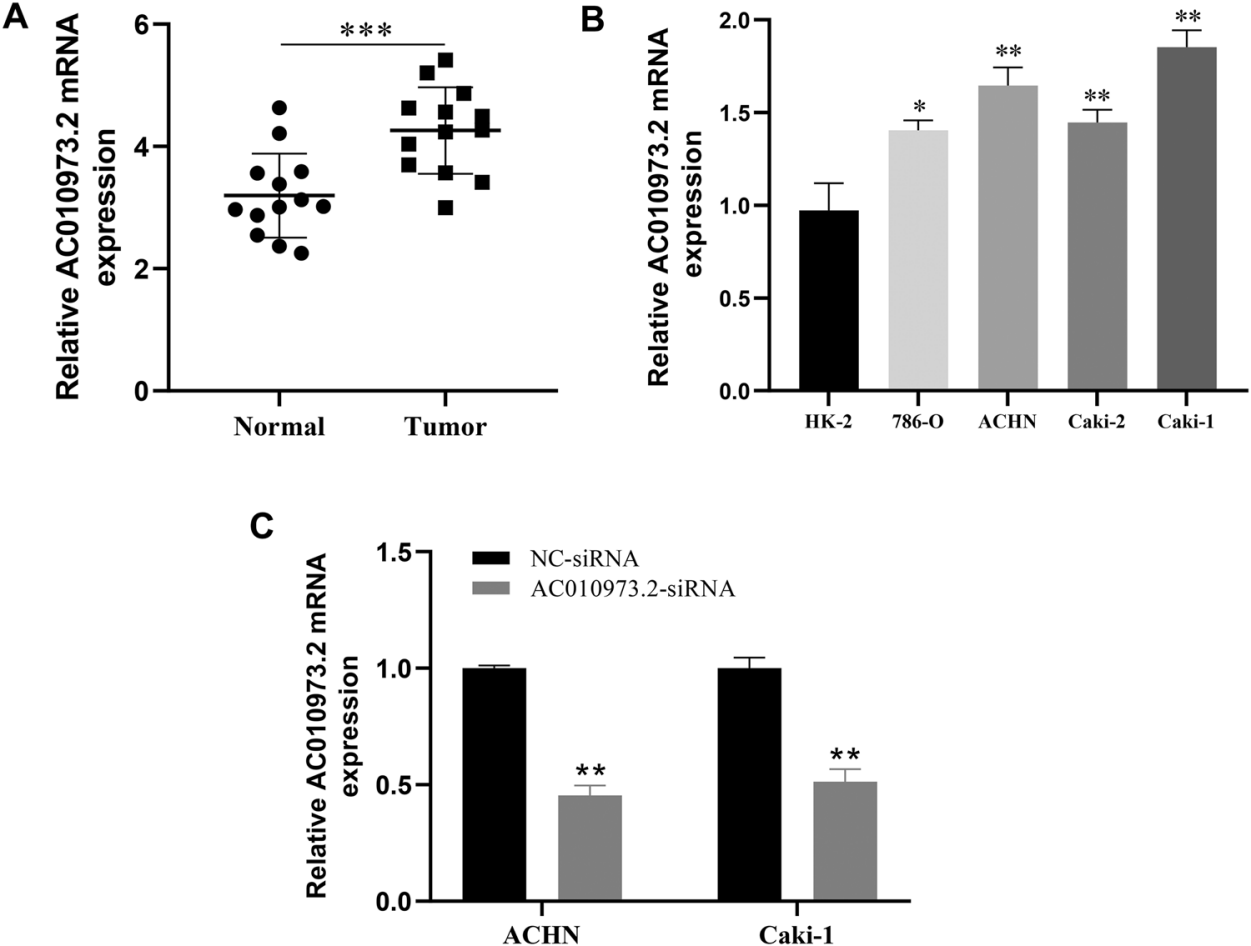
Upregulation of AC010973.2 mRNA expression in ccRCC. Notes: (**A**) AC010973.2 mRNA expression in tumors and non-malignant tissues from ccRCC patients. (**B**) AC010973.2 mRNA expression in ccRCC cell lines and normal renal tubular epithelial cells. (**C**) mRNA expression in AC010973.2 knockdown cell lines.

Based on 13 paired ccRCC tissue, qPCR results revealed prominent upregulation of *AC010973.2* mRNA expression in tumoral tissues (p < 0.01, Fig. 6A); this was also verified in cell level (Fig. 6B). *ACHN* and *Caki-1* were transfected with siRNA-*AC010973.2* for further research due to their highest expression level of *AC010973.2*. The knockdown of *AC010973.2* was confirmed by mRNA expression detection (Fig. 6C).

### *AC010973.2* promoted tumor proliferation through multiple oncogenic pathways in ccRCC

**Figure 7:**
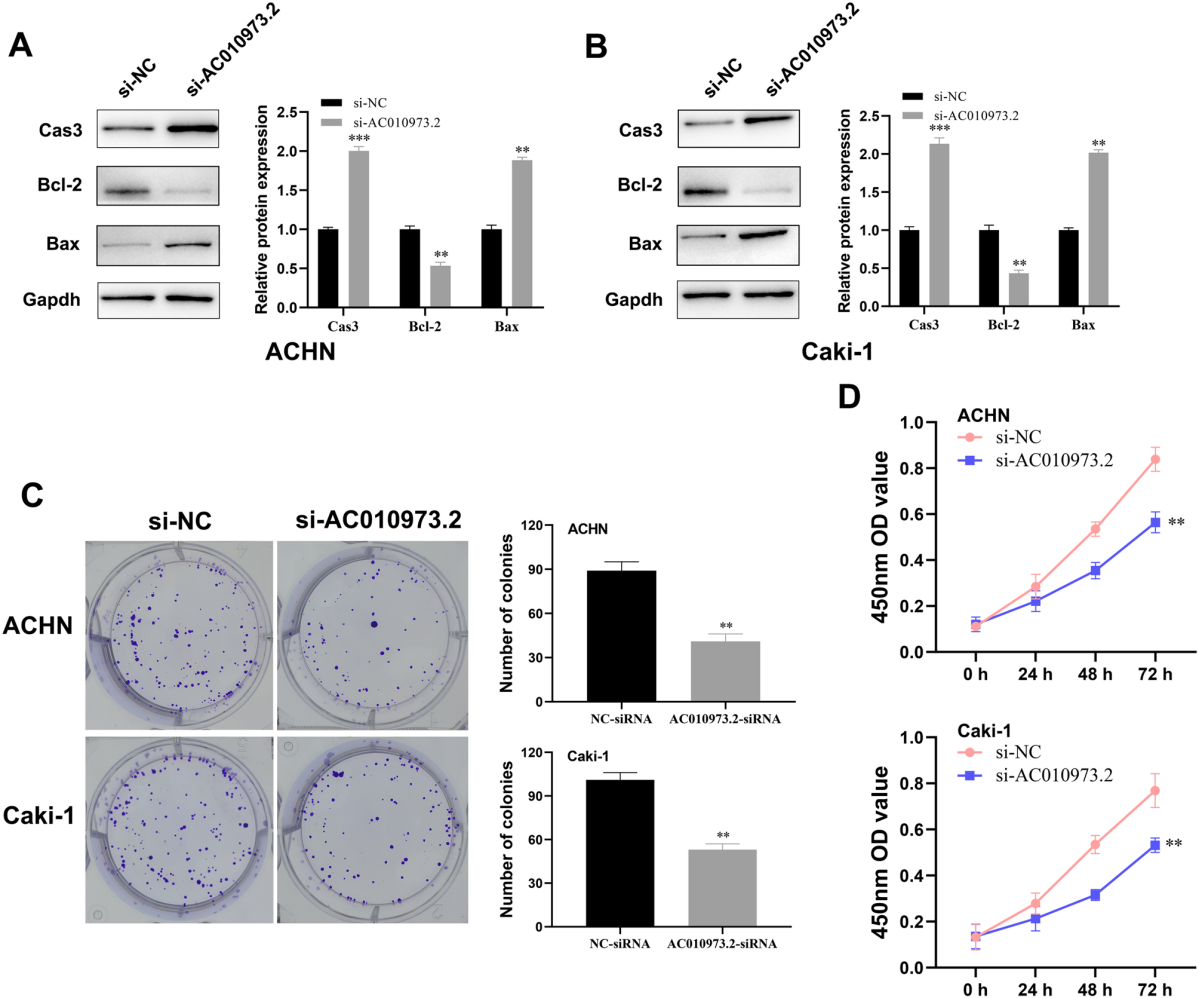
AC010973.2 regulated apoptosis signaling pathway and promoted cell proliferation. Notes: After the transfection with siRNA-AC010973.2, protein levels of Caspase-3, Bax and Bcl-2 in ACHN (**A**) and Caki-1 (**B**) cell lines through Western Blot; the blots were cut prior to hybridization with antibodies for saving antibodies. (**C**) Colony formation assay of ACHN and Caki-1 after the knockdown of AC010973.2. (**D**) CCK8 assay of ACHN and Caki-1 after the knockdown of AC010973.2.

Based on GSVA analysis, E2F targets, JAK/STAT3, G2/M checkpoints, apoptosis signaling pathways showed significant up-regulation in the high *AC010973.2* group (Fig. 5B). Notably, Caspase-3 acts as one of the most vital terminal operators of apoptosis in cells, and *Bcl-2* and *Bax* are pivotal regulators of *Cas-3*. After silencing *AC010973.2* in *ACHN* and *Caki-1* (Fig. 6C), the expression of the three proteins was detected by Western Blot. The protein expression of *Cas3* and *Bax* exhibited an apparent increase while *Bcl-2* was distinctly downregulated, both in *si-ACHN* and *si-Caki-1* cell lines, compared to *ACHN* and *Caki-1* cell lines (Fig. 7A-B).

Colony formation assays revealed that the colonies of the *si-AC010973.2* group were significantly less compared to that of the control group in both *ACHN* and *Caki-1* cell lines (Fig. 7C), suggesting that *AC010973.2* knockdown weakened the ability of tumor cells. Moreover, CCK8 assays demonstrated that *AC010973.2* knockdown profoundly inhibited the proliferation capacity of croc cell lines (Fig. 7D). Altogether, these results indicate that *AC010973.2* might promote tumor growth and survival by regulating multiple oncogenic pathways in ccRCC.

## Discussion

Renal clear cell carcinoma (ccRCC), specifically its metastatic form, cause high morbidity and mortality among renal cell carcinoma patients^1^. Tumor stemness-associated genes, small copies of tumor genes, promote tumor initiation, invasion, metastasis, and chemical resistance^14^. Several studies indicate that tumor stemness-associated cells are characterized by unlimited proliferation tendency and multiple differentiation potential, playing an essential vital role in developing multi-drug resistance and tumor recurrence^15,16^. Thus, sensitive and reliable prognostic biomarkers are essential in identifying patients with poor prognoses and who can benefit from treatment. This work evaluated the relationship between tumor stemness-associated genes and metastatic ccRCC. We developed a prognostic signature based on stemness-associated DEGs in ccRCC and successfully verified it in independent datasets using the Cox regression model. Further analysis revealed that the prognostic model constructed by 732 intersection genes demonstrated more advantages than clinical traits in predicting patient survival convenience. Eventually, colony formation assays showed that *AC010973.2* knockdown prominently inhibited the proliferation capacity of ccRCC cell lines. As a novel method predicting tumor prognosis, we confirmed the predictive effect of stemness-associated genes.

Chuan Liu et al. recently explored the relationship between immune-related gene pairs and cancer^17^. Nonetheless, limited studies have concentrated on the predictive value of stemness-associated genes. In the past few years, stemness-associated genes had been proposed as a novel sub-category gene and regulated tumor treatment of cancer^17^. The presence of malignant cells with stemness-associated is a significantly vital malignant feature of many tumors, showing important therapeutic significance^18^. These stemness-associated cells can self-renew and maintain tumor growth and appeared to be one of the primary reasons for tumor resistance, recurrence, and metastasis^19^. Interestingly, tumor stemness-associated genes also promote tumor metastasis in different tumor models and have been widely implicated in the ability to regulate proliferation behavior in various tumors^20^. Besides, one study found that stemness-associated cell behavior and malignant features are linked through epithelial-mesenchymal transition^21^. In addition to promoting stem cell differentiation, stemness-associated genes are related to tumor invasion and metastasis^22^. Here, stemness-associated genes promoted the progression and in ccRCC, and finally resulted in a poor prognosis.

Cancer stem cells, with the unique characteristics of self-renewing and generating allogeneic tumor cells, are considered to be the source of tumor progression, metastasis and recurrence. In recent decades, many researches have discovered that lncRNA is related to the stem cell phenotype of a variety of tumors. Hepatocellular carcinoma with stem characteristics is the key to tumorigenesis, chemotherapy resistance and progression. Zhang et al. revealed that Lnc-PDZD7 promotes stem cell characteristics and inhibits chemotherapy sensitivity through miR-101/EZH2/ATOH8 pathways, which provided new biomarkers for the diagnosis and potential drug targets of Hepatocellular carcinoma^23^. In addition, the functional studies have demonstrated that Lnc-FGF13-AS1 inhibits the proliferation, migration and invasion of breast cancer cell by impairing stem cell properties^24^. In this work, by using bioinformatics analysis, we establish a tumor stemness index related lncRNAs based prognostic prediction model, which involved AC010973.2, RNU6-125P, AP001209.2, Z98885.1, KDM5C-IT1, and AL021368.3, whose characteristics of stem cells that have not been reported in ccRCC before. It might provide a new direction for the future research on the interaction of lncRNAs on cancer stem cell characteristics.

Furthermore, we integrated the transcriptome profiling from the TCGA database and stemness-associated genes then conducted GO, KEGG enrichment, and Protein–protein interaction network analyses. Based on 732 intersection genes, random forest algorithms were used to model the prognosis of all 732 intersection genes, and finally, the top 6 important genes were selected for modeling. Consequently, the prognostic markers could be independently used to predict OS in ccRCC patients. In the univariate analysis, risk, grade, stage, pT, pM, and pN were related to the OS of ccRCC patients. Multivariate analysis revealed that pT and risk were associated with OS. We established a nomogram to predict the probability of survival at 1, 3, and 5 years by integrating critical clinical parameters and risk scores. As the most critical model gene, *AC010973.2* was selected for further research. The time-dependent ROC curve revealed the predictive value of *AC010973.2*. GSVA analysis demonstrated that some representative cancer pathways are closely related to *AC010973.2*, including E2F target, *IL6-JAK-STAT3* signaling, apoptosis signaling, hypoxia signaling, and fatty acid metabolism. In the validation part, qPCR results showed that the expression of *AC010973.2* mRNA in tumor tissues was significantly up-regulated. Notably, *AC010973.2* knockdown weakens the ability of tumor cells. Besides, CCK8 analysis showed that *AC010973.2* knockdown could significantly hinder the proliferation of ccRCC cell lines.

## Conclusion

In conclusion, we investigated the biological functions and prognostic value of stemness-associated genes in ccRCC. As a consequence, bioinformatics analysis revealed that stemness-associated genes potentially regulate the apoptotic signaling pathway, influencing tumor progression. Besides, the prognostic signature of stemness-associated genes is potential diagnostic and prognostic biomarkers in ccRCC. Furthermore, we identified *AC010973.2* for further research, where, in vitro experiments revealed that *AC010973.2* could significantly promote cell proliferation of ccRCC and might be a potential prognostic marker.

## Supplementary Information

Supplementary Information.
