# Supplementary figures and images for: AC010973.2 promotes cell proliferation and is one of six stemness-related genes that predict overall survival of renal clear cell carcinoma
Source: Sci Rep. 2022 Mar 11;12:4272. doi: 10.1038/s41598-022-07070-1 (PMC8917182; doi:10.1038/s41598-022-07070-1)

GAPDH


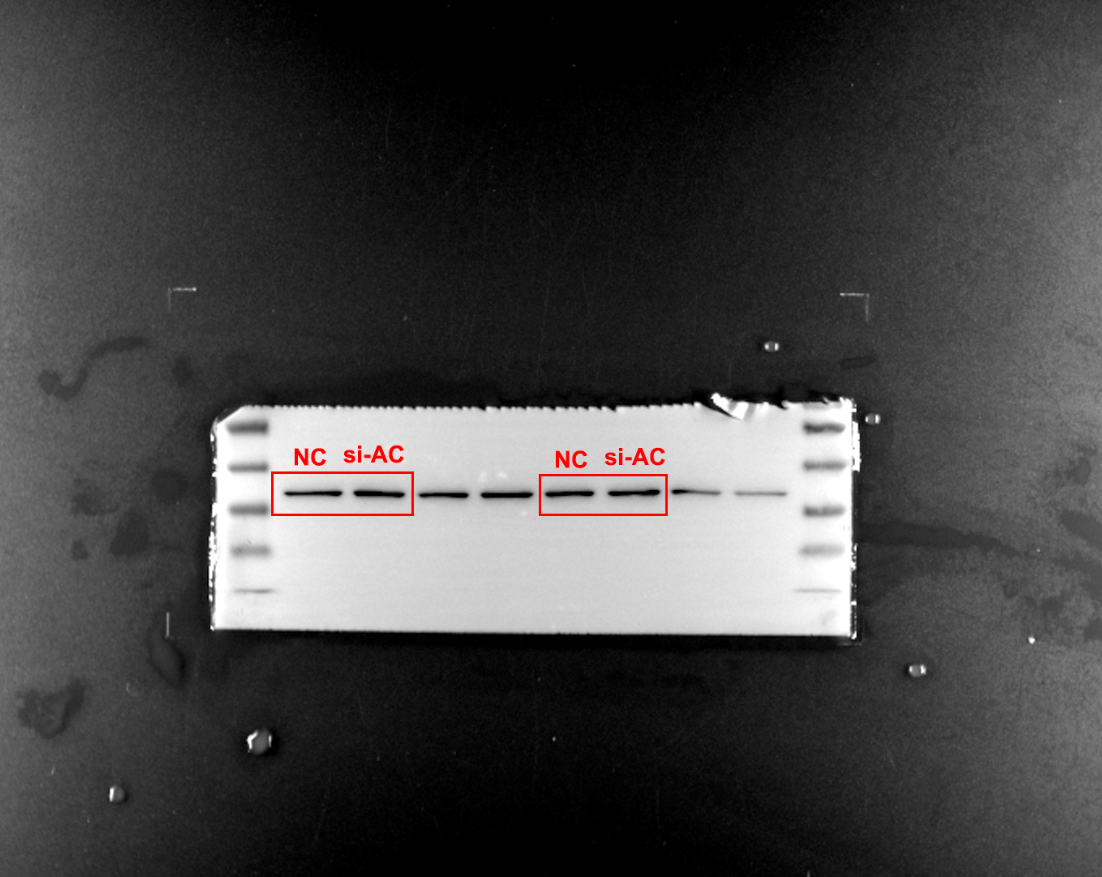


Bax


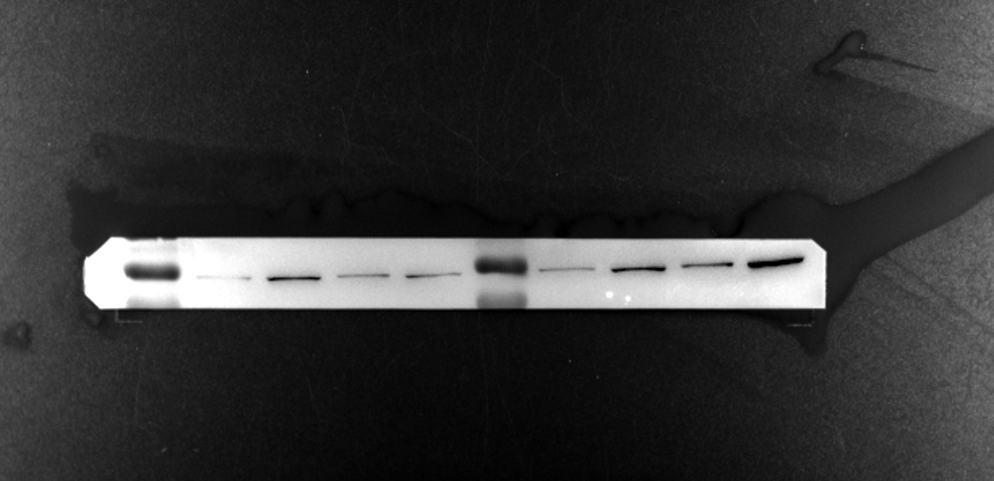

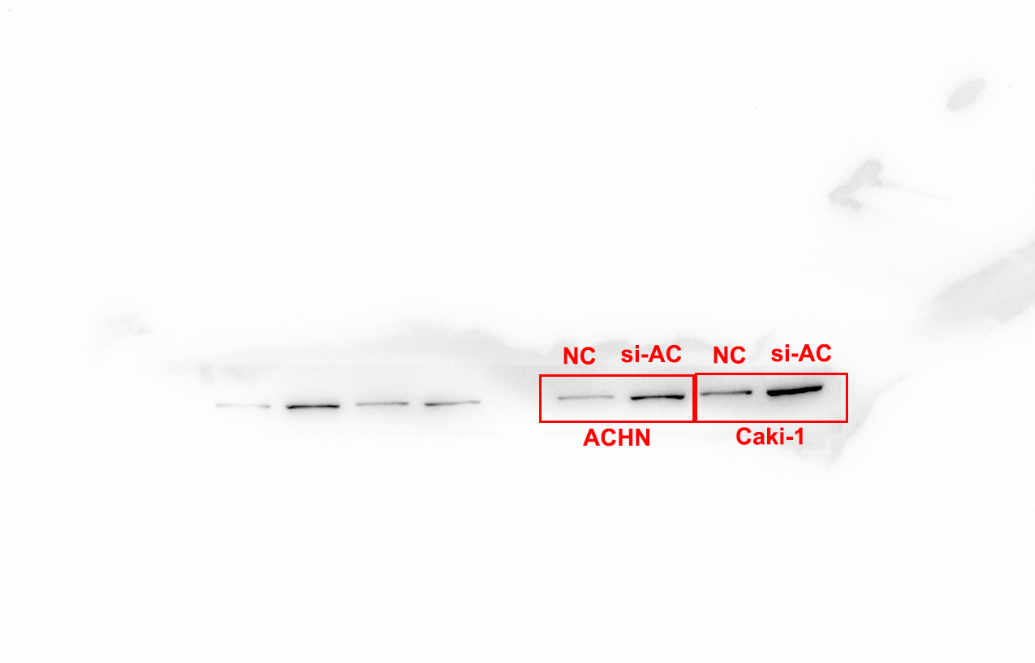


Bcl-2


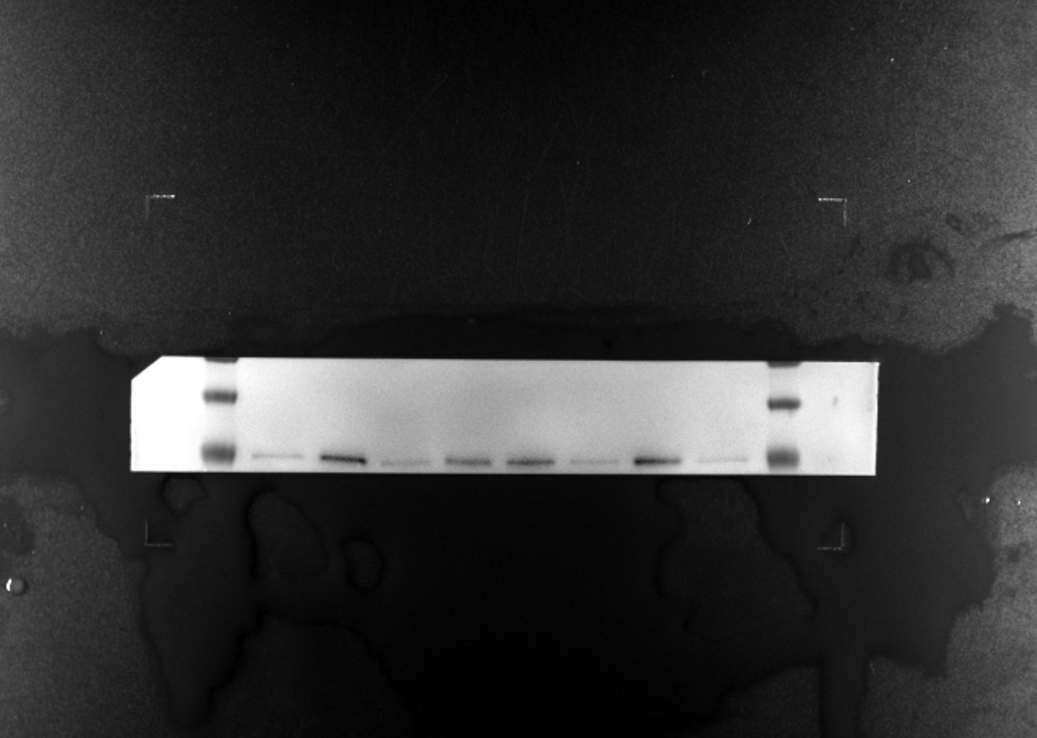


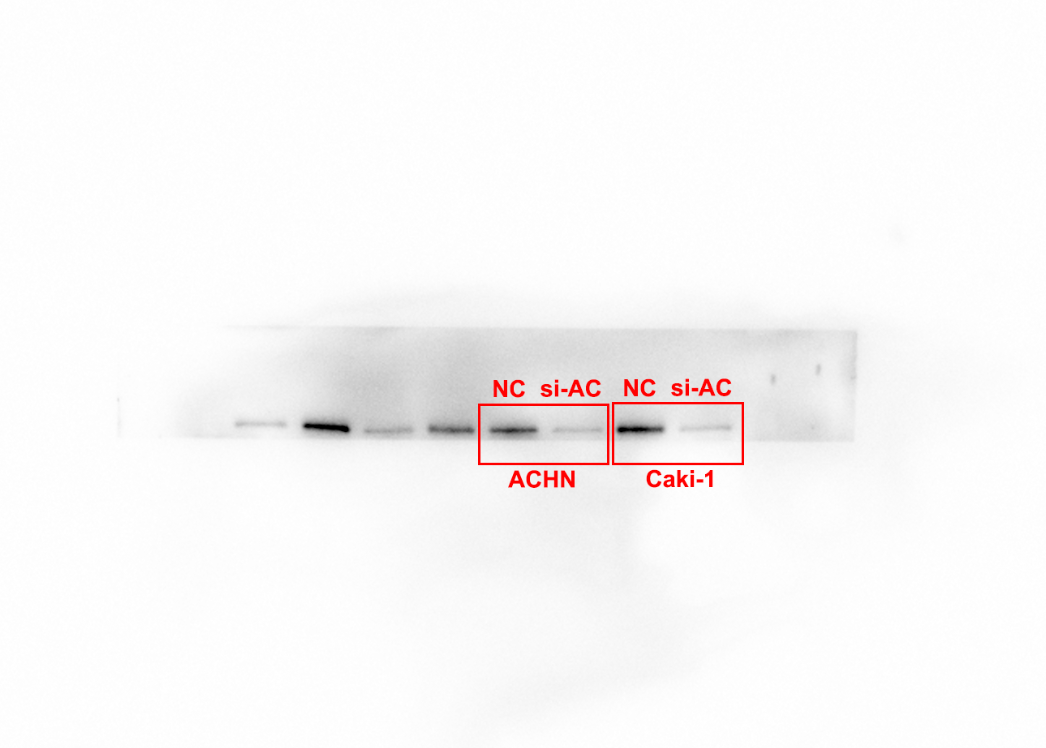


Cas3


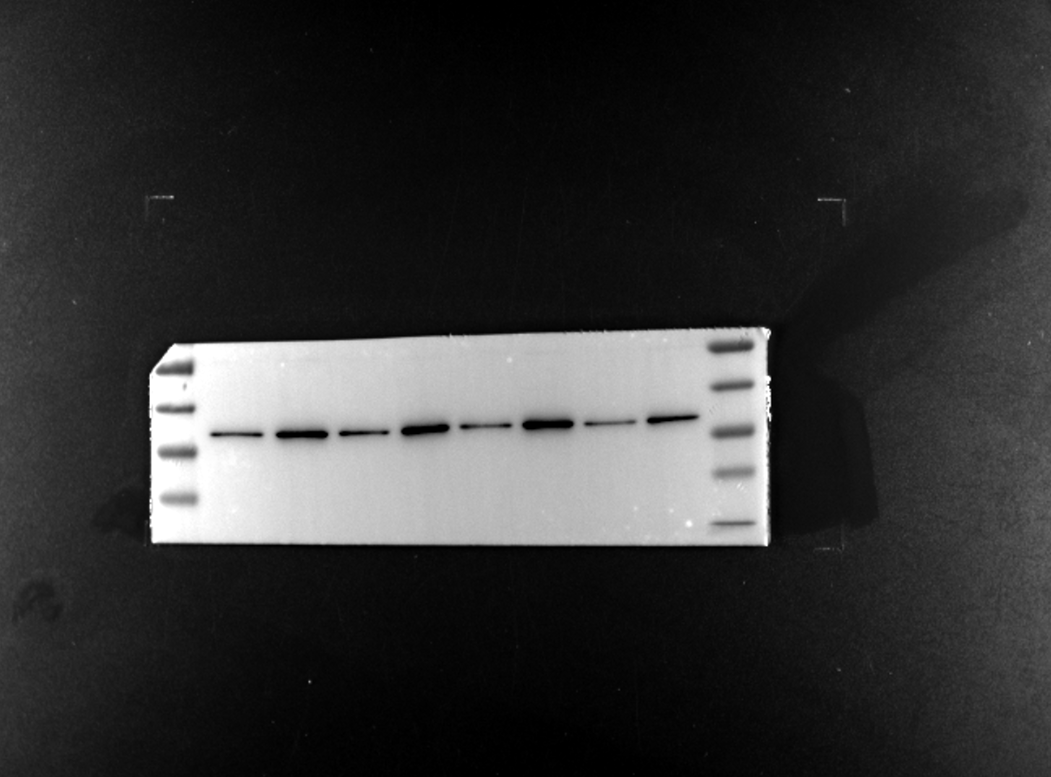


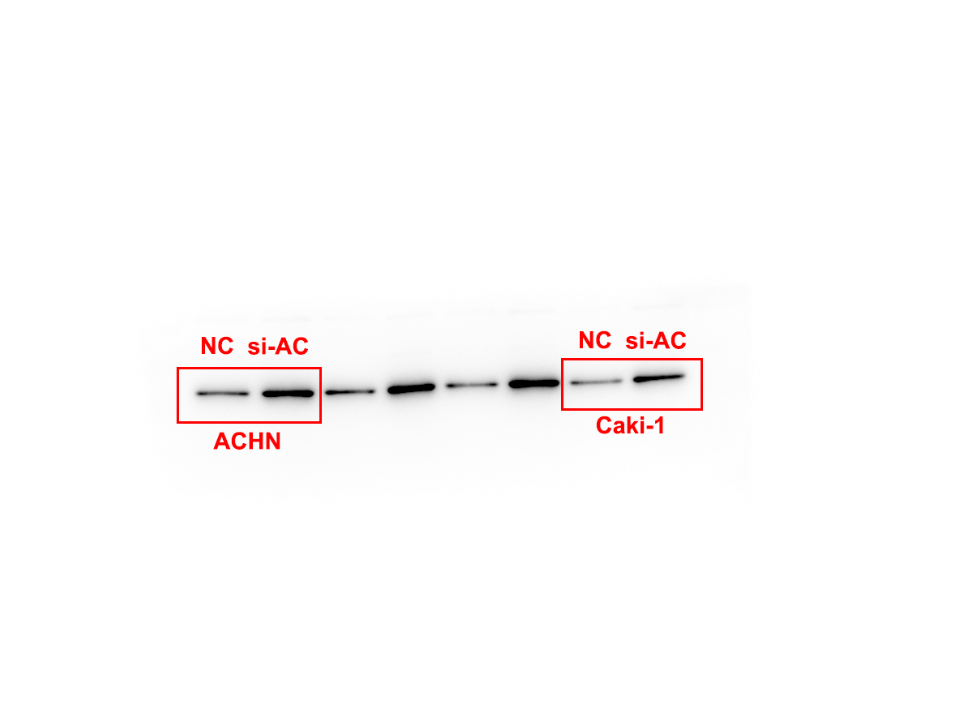

Supplement: Supplementary file 1 — Supplementary Information. [file 41598_2022_7070_MOESM1_ESM.docx]
